# Platelets Proteomic Profiles of Acute Ischemic Stroke Patients

**DOI:** 10.1371/journal.pone.0158287

**Published:** 2016-06-23

**Authors:** Ozge Cevik, Ahmet Tarik Baykal, Azize Sener

**Affiliations:** 1 Cumhuriyet University, Faculty of Pharmacy, Department of Biochemistry, Sivas, Turkey; 2 Acibadem University, School of Medicine, Department of Medical Biochemistry, Istanbul, Turkey; 3 Marmara University, Faculty of Pharmacy, Department of Biochemistry, Istanbul, Turkey; Universität Regensburg, GERMANY

## Abstract

Platelets play a crucial role in the pathogenesis of stroke and antiplatelet agents exist for its treatment and prevention. Through the use of LC-MS based protein expression profiling, platelets from stroke patients were analyzed and then correlated with the proteomic analyses results in the context of this disease. This study was based on patients who post ischemic stroke were admitted to hospital and had venous blood drawn within 24 hrs of the incidence. Label-free protein expression analyses of the platelets’ tryptic digest was performed in triplicate on a UPLC-ESI-qTOF-MS/MS system and ProteinLynx Global Server (v2.5, Waters) was used for tandem mass data extraction. The peptide sequences were searched against the reviewed homo sapiens database (www.uniprot.org) and the quantitation of protein variation was achieved through Progenesis LC-MS software (V4.0, Nonlinear Dynamics). These Label-free differential proteomics analysis of platelets ensured that 500 proteins were identified and 83 of these proteins were found to be statistically significant. The differentially expressed proteins are involved in various processes such as inflammatory response, cellular movement, immune cell trafficking, cell-to-cell signaling and interaction, hematological system development and function and nucleic acid metabolism. The expressions of myeloperoxidase, arachidonate 12-Lipoxygenase and histidine-rich glycoprotein are involved in cellular metabolic processes, crk-like protein and ras homolog gene family member A involved in cell signaling with vitronectin, thrombospondin 1, Integrin alpha 2b, and integrin beta 3 involved in cell adhesion. Apolipoprotein H, immunoglobulin heavy constant gamma 1 and immunoglobulin heavy constant gamma 3 are involved in structural, apolipoprotein A-I, and alpha-1-microglobulin/bikunin precursor is involved in transport, complement component 3 and clusterin is involved in immunity proteins as has been discussed. Our data provides an insight into the proteins that are involved in the platelets’ activation response during ischemic stroke. It could be argued that this study lays the foundation for future mechanistic studies.

## Introduction

Ischemic stroke is a serious disease that results in high morbidity and mortality rates. Stroke is caused by vascular blockages in the brain that cripples blood flow. [1]. Platelets play a crucial role in the pathogenesis of stroke [2,3] and its treatment through prevention is possible through antiplatelet agents [4]. Antiplatelet agents can suppress the platelet activation and aggregation, thereby eliminating the high atherosclerosis risk of stroke patients. In the event of antiplatelet therapy ceasing, the risk of stroke may rise in high-risk patients [5–7]. On the other hand, the activation of platelets occurs via vascular subendothelium, collagen, fibrin, tissue factor and shear stress which are stimulants in the blood circulation [8]. Platelets can be affected by various conditions in the circulation and such variations can trigger the cascade of thrombus processes [9]. Platelets generate microparticles which upon activation exhibit various pathophysiologic functions like the initiation and exacerbation of stroke [10]. Platelet activation markers are associated with the platelet surface during activation such as PAC-1 (Procaspase Activating Compound-1) CD62P (P-selectin), CD31 (PECAM, Platelet endothelial cell adhesion molecule) and CD63. Glycoprotein 2b/3a (Gp2b/3a) (also known as integrin αIIbβ3) is a major membrane receptor and plays an essential role in calcium dependent platelet adhesion and activation. PAC-I, a monoclonal antibody, is a marker for platelet activation that binds only to the stimulated Gp 2b/3a complex on activated platelets and not to those resting. When Gp2b/3a activation is initiated platelets undergo conformational and surface changes that enhance their affinity to bind to fibrinogen, resulting in platelet-platelet adhesion, aggregation and platelet-leukocyte interaction [11]. In particular, Flow cytometry detection of PAC-I is more clinically applicable in the evaluation of platelet function [12]. Gp2b/3a which is the platelet activation receptor can be a target for therapeutic drugs and such Gp2b/3a inhibitors are specifically used to halt this activation in platelets [13–15]. It has been reported that the Gp2b/3a receptor is a target for anti-platelet therapy in the stroke model in mice [16,17].

When the issue of an effective anti-platelet therapy for stroke patient is considered, platelet functions and cellular components need to be investigated and determined. Due to the fact that stroke is associated with the formation of thrombus it was deemed imperative for the protein expressional changes in the platelets of stroke patients to be investigated. Our research based on the scope of this study has not encountered another such proteomic study in the literature of the field, thereby rendering the current study the first of its kind.

## Material and Methods

### Participants

This study was approved by the Human Research Ethics Committee of Marmara University and was conducted according to the Declaration of Helsinki. This study required that all patients and control subjects signed an informed constant form prior to blood sampling. Platelet samples were obtained from patients who had suffered a stroke and were admitted to the neurology department within 24hrs of the incident and had not yet been administered with any medication.

The diagnosis was made in accordance with the criteria of the World Health Organization where stroke is defined as rapidly developing clinical symptoms/signs of cerebral dysfunction lasting more than 24 hours without any cause other than a vascular abnormality. The stroke subtype of the 65 patients who were admitted (mean age 66.1±10.5 years, 45–75 years old) was classified according to the TOAST (trial of Org 10172 in acute stroke treatment) classification. Exclusion criteria included transient ischemic attack (TIA), hemorrhagic stroke, intracerebral hemorrhage and cranial trauma. The control group consisted of 42 participants (mean age 57.9±10.2 years, 40–68 years old) who underwent evaluation in the internal medicine polyclinic for routine controls. The control group comprised of subjects who hadn’t taken anti-platelet drugs over the last 14 day period. Prior to the drawing of venous blood a record of prescription medication used by subjects was made. In addition, the demographic characteristics of sampled subjects are shown in S1 Table.

### Platelet preparation

Human blood was collected into tubes containing acid citrate dextrose (364606 BD, USA) and platelet-rich plasma (PRP) was prepared via centrifugation at 150 g for 15 minutes at room temperature. PRP was then recentrifuged at 800 g for 15 minutes to concentrate the platelets and the pellet was resuspended in modified Tyrode’s Ca^+2^/Mg^+2^ free buffer (127 mM NaCl, 2.7 mM KCl, 0.5 mM NaH_2_PO_4_, 12 mM NaHCO_3_, 5 mM 4-(2-hydroxyethyl)-1-piperazineethanesulfonic acid (HEPES), 5.6 mM glucose, pH: 7.4) in order to obtain a final platelet concentration of 2x10^8^/mL.

### Platelet PAC-I binding assay

In order to determine platelet activation, PAC-I (activated Gp2b/3a receptor marker) binding was measured using flow cytometry. Platelet suspensions were incubated with 5 μg/mL FITC-labeled anti-PAC1 (BD, USA) into a polypropylene tube containing PBS and 1% BSA for 30 minutes at room temperature. The samples were subsequently diluted with buffer and analyzed through flow cytometry.

### Platelet calcium secretion measurement

Platelet intracellular calcium flux was measured with a Fluo-8 calcium assay kit (AAT Bioquest). Fluo-8 solution and dye-loading solution was added to the platelet suspensions and platelet cells were incubated for 30 min at 37°C. The Ca^2+^ level was determined by monitoring the fluorescence intensity in Ex/Em = 490/525 nm with a fluorescence microplate reader (Glomax, Promega, USA).

### Western blot analysis

Platelet lysates were suspended in cell lysis buffer with a protease inhibitor cocktail and centrifuged for 15 minutes at 14.000 rpm, at +4°C. The supernatant was collected and the protein concentration was determined using the Bradford method [18]. Once the proteins had resolved on 4–12% SDS-PAGE and were transferred to a nitrocellulose membrane and blocking achieved with BSA, the gel was incubated overnight with the primary antibody (1:500 monoclonal human anti-Gp2b/3a sc-53417, b-actin sc-130301 Santa Cruz Biotechnology). The membrane was then washed and incubated with HRP conjugated secondary antibody for 2 hours and the blot was developed with chemiluminescence reagents and then exposed to the film. The quantification of band intensities was realized through Image J analysis software (NIH, Bethesda, MD). Band intensities were normalized to the corresponding controls and shown as the mean ±SD. A representative blot for each protein of interest is shown, with β-actin as loading control.

### Proteomics analysis by LC-MS

#### Sample preparation

In selected stroke patients (n = 9) and control subjects (n = 9) platelets were rinsed first with cold 50 mm ammonium bicarbonate (AmBic) and protein extraction was achieved with an ultrasonic homogenizer (5 s on, 5 s off, 3 cycles). The realization of the protein concentration of the cell lysate was based on the Bradford method [18]. For the tryptic peptide generation sample analyses, 100 ug of total protein mixture was used. The filter aided sample preparation (FASP) method was applied to generate the tryptic peptides where 200 ul of 6 M urea was added to the protein sample and centrifuged at 14,000 x g for 15 in a 30 kDa cut-off spin filter. Protein mixture was alkylated with 10 mM iodoacetamide in the dark at room temperature for 20 minutes and then rinsed first with 6 M urea and then twice with 50 mM AmBic solution. The mixture was incubated with 1:100 (trypsin: protein) ratio of MS grade trypsin overnight. Later the peptides were removed from the spin filter with 50 mM AmBic and 0.5 M NaCl solution. The flow- through was collected and lyophilized.

#### LC-MS/MS Analysis and Database Search (UPLC-ESI-qTOF-MS)

LC-MS/MS based protein expression analysis was carried out in accordance with our prior research [19]. In brief, 500 ng total tryptic peptide mixture was analyzed in triplicate by a LC-MS/MS system (nanoACQUITY UPLC and SYNAPT G1 HDMS, Waters). The trap and analytical columns were equilibrated with mobile phase (97% H_2_O with 0.1% formic acid and 3% ACN (Acetonitrile) with 0.1% formic acid) and column temperature was 45°C. The gradient elution of peptides was achieved by increasing the ACN percentage from 5 to 40% at a 300 nl/min flow rate over a 90 minute period. Symmetry C18 5 μm, 180 μm i.d. x 20 mm and BEH C18, 1.7 μm, 75 μm i.d. x 250 mm columns were employed as both trap and analytical columns respectively. MS^E^ data was collected at positive ion V mode with 1,5 second intervals switching from 6V low energy to ramped collusion energy from 15 to 40 V. Glu-fibrinopeptide was infused as a mass calibrant and peptide signals between 50–1600 were recorded. Signal preprocessing was undertaken with ProteinLynx Global Server (v2.5, Waters) and peptide sequences were searched against the reviewed database of homo sapiens (www.uniprot.org). The sequence of the internal standard (yeast enolase, Uniprot accession no: P00924) was included in the FASTA file. The level of Carbamidomethyl-cysteine was fixed and Acetyl N-TERM, the deamination of asparagine and glutamine and oxidation of methionine, underwent variable modifications. The Apex3D data preparation parameters were set in accordance with o previous research [20, 21].

#### Protein Identification and Quantification

The absolute quantification of proteins is achieved through the addition of a known amount of internal calibrant to the tryptic peptide mixture. Three of the most intense peptides of the added calibrant are used to calculate the absolute amounts of each protein within the mixture. Three of the reproducibly detected calibrant peptides are m/z 1286.7098, m/z 1046.5273 and m/z 1159.6082 which are used to calculate an average response. Each sample that will undergo analysis is spiked with 50 fmol of calibrant. For the purpose of quantitation, three of the most intense peptide signals from a protein are selected and compared to the intensities calculated for the internal calibrant and absolute amounts are then derived [22–24]. Progenesis LC-MS (V4.0, Nonlinear Dynamics) was used to calculate the normalized abundances for all of the identified proteins.

### Statistical Analyses

Statistical computations for biochemical analyses were performed using SPSS software package version 15.0 (SPSS). Data normality was assessed using the Kolmogorov–Smirnov test. Results are presented as mean ± standard deviation as appropriate. The Mann–Whitney U-test was applied to assess the differences in biomarker concentration between the 2 groups.

## Results

### Platelet activation in stroke patients

**Fig 1:**
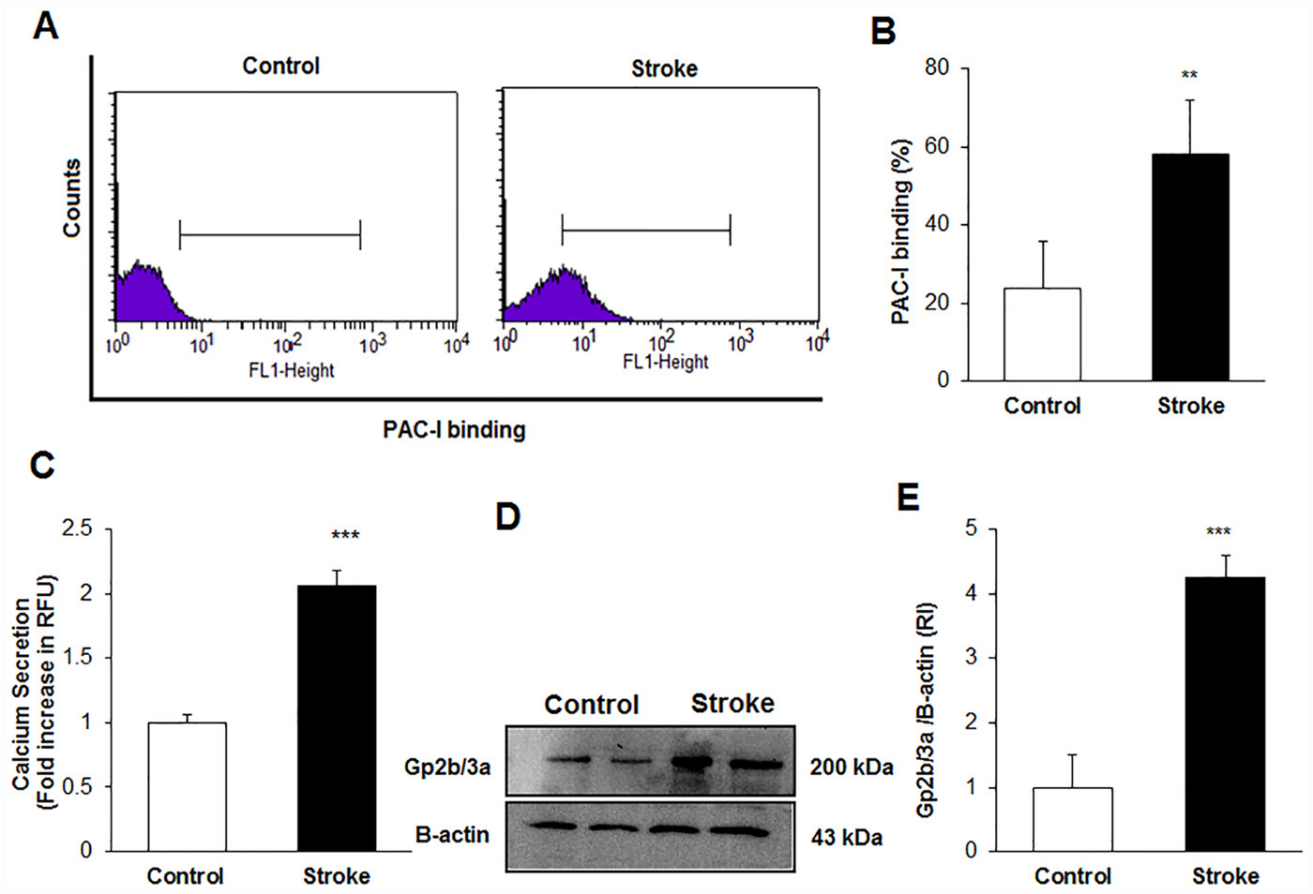
**A)** Histogram of Platelet activation responses using PAC-I binding by flow cytometry in the control and stroke patient groups. **B)** The percentage of PAC-I binding in the stroke and control subjects (**p < 0.01 versus control group) **C)** Platelet Ca^+2^ levels secretion in the stroke and control subjects (**p < 0.001 versus control group) **D)** Gp2b/3a protein expression in platelets determined by Western blotting. **E)** Gp2b/3a/β-actin ratio in the platelets (***p<0.01 versus control group)

Platelet histograms of PAC-I binding, as a marker of activation of Gp2b/3a receptor, for both control subjects and stroke patients are shown in Fig 1A. When the parameters in the control group (24.28 ± 12.04%) were compared with those in the stroke group (58.79±14.15%), it was found that the PAC-I binding levels in the stroke patients were significantly higher than the controls (Fig 1B, p<0.01). Platelet Ca^2+^ secretion, an activation and aggregation marker, is shown in Fig 1 for the samples. The use of Ca^2+^ specific binding fluorescence particles against platelets Ca^2+^ secretion was measured by a fluorescence reader (Fig 1C). Ca^2+^ secretion ratio levels of stroke subjects (2.06 ± 0.12) were found to be significantly higher than the control group (1.04 ± 0.06, p<0.001). The detection and quantification of the platelet activation marker protein, Gp2b/3a, was achieved by western blotting (Fig 1D). Gp2b/3a protein was detected in the membrane fraction as a 200 kDa band and β-actin is seen as a 43 kDa band. When the Gp2b/3a protein expression values of the control subjects (1.05 ± 0.81) were compared to stroke subjects (4.25 ± 0.38), significant increases were observed (p<0.001, Fig 1E). The data indicate that the activation stimulus is persistent in stroke patients.

### Proteomic profiling of platelets

A total of 500 unique proteins in stroke patients were identified and grouped according to the appropriate refseq IDs (a complete list of all identified proteins is shown in supplementary data 2). The proteins which had a higher or lower expression pattern in stroke patients were then ascertained. It was found that eighty-three proteins are differentially expressed in the stroke patient when compared to the control patient. Protein localization, classification, fold change and significances are also shown in S2 Table.

According to the Gene Ontology database the statistically significant proteins identified are localized in terms of percentage as 36% in the cell cytoplasm, 42% in the extra cellular space, 11% in the membrane, 3% in the cytoskeleton and 8% is unknown localization (S1 Fig). Furthermore, the same list of proteins were grouped according to their biological processes and it was found that 17% were involved in the cellular process, 16% in biological regulation, 11% in localization, 12% in response to stimulus, 10% in the multicellular organismal process, 8% in metabolic process, 7% in the establishment of localization and 19% in the other pathways (S2A Fig). The direct and indirect simultaneous interaction of the proteins was investigated. Of the proteins identified 33% were categorized as playing a role in molecular function, 32% in binding, 13% in catalytic activity, 4% in structural molecule activity and 18% fell in other categories (S2B Fig).

**Fig 2:**
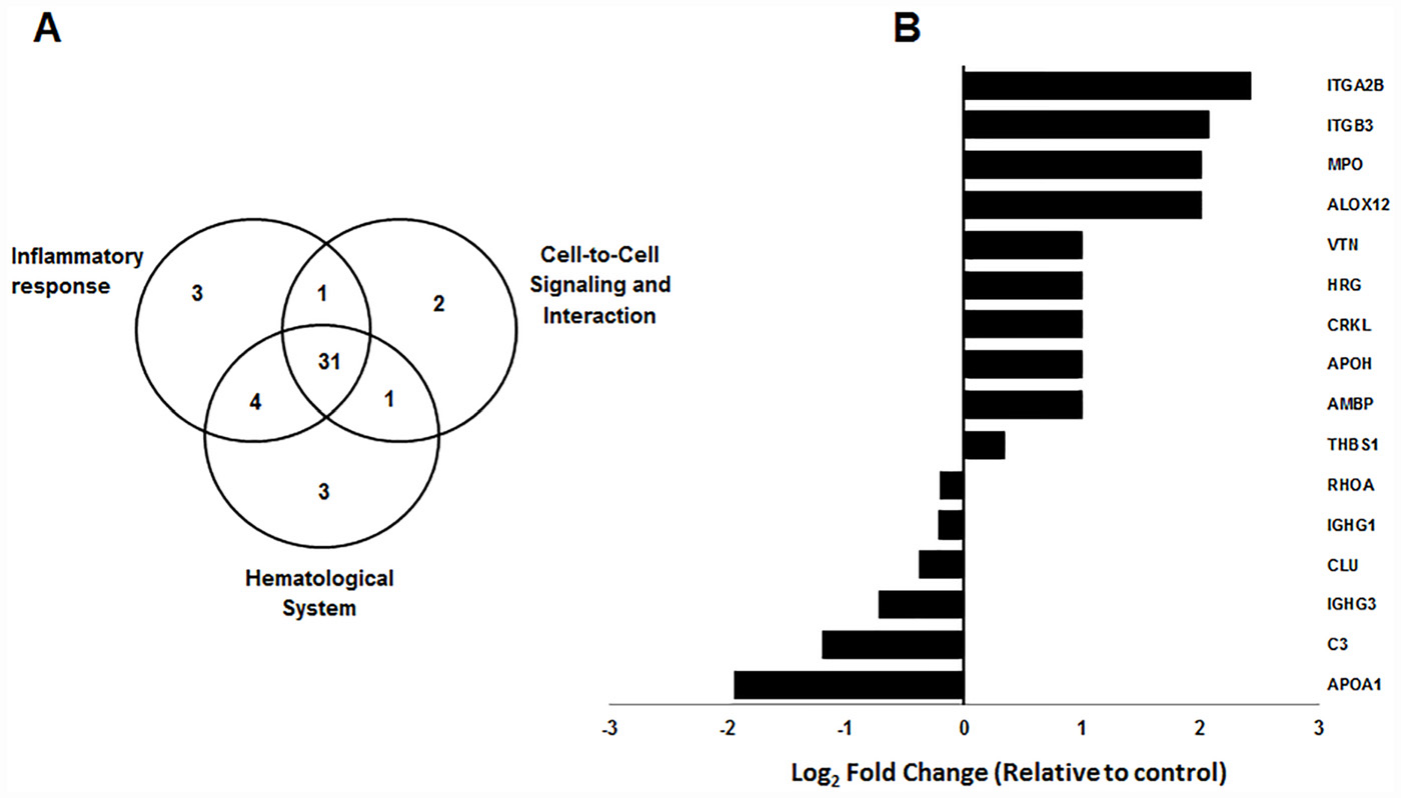
**A)** Venn diagram indicating the overlap between the differentially expressed proteins identified in stroke patients with respect to control group. **B)** Contribution of differentially expressed proteins in stroke patients in comparison to control group.

Protein-protein interaction networks were generated using the IPA (Ingenuity Pathway Analysis) software and based on the proteins identified; it was found that the statistically significantly altered proteins play a role in cell-to-cell signaling and interaction, inflammatory response, immune cell trafficking, cellular movement, hematological system development and function, and nucleic acid metabolism. Functional analyses also showed that certain pathways are overrepresented in this set which includes hematological, cell-to-cell signaling and inflammatory diseases. As shown in Fig 2A the patterns of change are consistent in both directions as is the magnitude of the significance in these three pathways. In terms of the proteins up-regulated in stroke patients, 39 of the proteins played a role in inflammatory response, 35 proteins in cell-to-cell signaling and interaction and 39 proteins in the hematological system On the other hand, these three processes were constructed from 31 proteins and thirty-one of the 83 proteins could be matched and appeared on all three pathways. However, the expression difference levels for these 16 were significantly higher (as shown in in Fig 2B) and these proteins are represented in all three pathway.

## Discussion

In the present study, it was first determined that the platelet activation of ischemic stroke patients resulted in altered protein expressions. Various platelet abnormalities are implicated in atherothrombotic mechanisms in ischemic stroke and to clinically alleviate it, anti-platelet drugs and anticoagulants are prescribed [25,7]. Platelets can interact highly with other cells in the circulation owing to their high concentration and reactivity.

Structural or molecular changes in the cell membrane result in a direct or indirect cellular response. Platelets have short life spans which are more prone to changes in plasma molecules and cell interactions in blood circulation. Upon activation, the cellular component organization of platelets can be altered and molecular interaction also stimulates neighbouring cells[26]. Platelet adhesion and activation leads to thrombus formation which reduces the risk of stroke [6]. It was previously demonstrated that the high plasma levels of TNF-α (tumor necrosis factor alpha) stimulated platelet activation in stroke patients [27]. Therefore, anti-platelet drugs or combination therapies are utilized for the prevention of stroke. This study witnessed the analysis of the proteomic profiles of platelets from acute ischemic stroke patients. The results of platelet activation studies demonstrated that platelets from stroke patients caused a significant increase in PAC-I binding, Gp2b/3a activation and Ca^2+^ secretion. Previous studies demonstrated that platelet activation involving PAC-I binding enhances the acute phase and returns to baseline three months later in acute ischemic stroke patients [28]. Similarly anti-platelet combination therapies decrease the expression of PAC-I in platelets of stroke patients [29].

The findings of this study determined that several important proteins like cellular metabolic process proteins MPO (Myeloperoxidase), ALOX12 (Arachidonate 12-Lipoxygenase), HRG (Histidine-rich glycoprotein), cell signaling proteins CRKL (Crk-like protein), RHOA (Ras homolog gene family, member A), cell adhesion proteins VTN (Vitronectin), THBS1 (Thrombospondin 1), ITGA2B (Integrin alpha 2b), ITGB3 (integrin, beta 3), structural proteins APOH (Apolipoprotein H), IGHG1 (Immunoglobulin heavy constant gamma 1), IGHG3 (Immunoglobulin heavy constant gamma 3), transport proteins APOA1 (apolipoprotein A-I), AMBP (Alpha-1-microglobulin/bikunin precursor) and immunity proteins C3 (complement component 3), CLU (Clusterin) were present in platelets of stroke patients. These proteins may be potential targets for early stage stroke.

ITGA2B and ITGB3 receptor complex of the integrin family is encoded in Gp2b/3a (CD41/CD61) and is expressed on platelets and megakaryocytes that play a critical role in thrombosis and hemostasis. Adenosine diphosphate activates Gp2b/3a which can lead to platelet stimulation signaling pathways [30]. As highlighted in the results section, the Gp2b/3a complex protein expressions of platelets were quantified using western blotting in stroke patients. The platelet proteomic profiling data revealed that the differences in log2 ratio intensities between ITGA2B and ITGB3 levels of stroke patients and of those patients in the control group. These data enable comparison of platelet activation and platelets surface receptors levels between stroke patients and healthy patients. VTN is a major cell adhesion multifunctional glycoprotein and is released to alpha granules from platelets that play a role in the formation of stable platelet aggregates [31, 32]. In addition, VTN interactions with platelets integrin Gp2b/3a triggers VTN dependent platelets adhesion that can lead to thrombus formation of atherosclerosis [33,34]. It was found that VTN protein expression is higher in stroke patients’ platelets. The other glycoprotein is HRG which has been identified binding to activated human platelets [35] and includes zinc, haem and calcium ion binding site [36]. HRG can bind activated platelets [37]. According to the findings, HRG binding on platelet surface can occur due to increased platelet activation during a stroke. CRKL is an adaptor protein that is essential for the tyrosine kinase signaling pathway [38]. CRKL has a role in platelet aggregation through inducing tyrosine phosphorylated via thrombopoietin in both chronic myeloid leukemia patients’ [39] and upregulated in myocardial infraction patients’ platelets [40]. Furthermore, CRKL protein has an association and shows a functional response after stimulation of the collagen receptor GPVI in platelets [41]. In the present study, an increase in CRKL protein expression was also seen in stroke patients’ platelets in response to platelet activation. In addition to this, THBS1 also plays a role in cell-cell, cell-matrix interactions, platelet aggregation and angiogenesis which is stored in the α-granules of platelets and stimulates Gp2b/3a dependent platelet activation [42]. It has been shown that THBS1 constitutes a sensitive and stable parameter suited to monitoring in vitro platelet activation [43]. Likewise, the mechanism by which THBS1 contributes to up regulation after stroke in animal models [44].

AMBP is a two plasma glycoprotein and combines with α-1-microglobulin and bikunin precursors that play a role in the regulation of inflammatory processes [45]. Recent studies show that AMBP may be a marker for differential diagnosis such as bowel disease [46], cancer [47] and kidney stones [48]. However, there is currently little information with regard to platelets in the literature of the field. RHOA is a member of the Ras superfamily of small GTP-binding proteins which plays a highly selective role in Gp2b/3a signaling in platelets [49]. It has a role in response to integrin ligation that maintains platelet sphericity and promotes contractility [50]. Recent studies reveal that RHOA is activated during the initial phase of platelet activation but is inactivated during the spreading and late phase of activation [51]. At the same time, it is reported that RHOA deficiency affects platelet function and the structure of microtubules which is the potential association of RHOA to deregulation in human hematologic diseases [52]. Interestingly this deficiently has been associated with stroke patients’ platelets activation and expression of Gp2b/3a.

CLU is a glycoprotein that has a functional role in pro-survival and cell cycle control [53]. Ordinarily, it is the platelets and megakaryocytes that expressed the CLU which is localized in the alpha granules [54]. It is reported that CLU protein expression decreased in systemic lupus erythematosus patients’ platelets and interacted with thrombotic complications [55]. Lowered CLU levels in platelets may be involved in the pathogenesis of stroke patients.

Another important consideration is that complement-cell interactions may be receptor-dependent or independent. Proliferation, cell death, mitosis, cell arrest, secretion and phagocytosis are some of the many cellular responses that can be triggered by these complements [56]. The activation of complements is directly linked to inflammation which is the major cause of autoimmune and immune complex diseases [57]. Complement C3 binds to activated platelets, the presence of complement proteins on the surfaces of non-activated and thrombin receptor-activating peptide-activated platelets is revealed by flow cytometry [58]. This is in contrary to a number of pathological conditions where thrombocytopenia and thrombotic diseases occur without the regulators of complement activation. Likewise, IGHG1 and IGHG3 proteins correlated to an immunological and inflammatory pathway [59] and the role of such proteins function is little discussed in the literature of the field. Inflammation markers have been associated with the early phase of stroke in the activation of innate local immune responses in glial cells and the recruitment of circulating leukocytes into the affected brain tissue [60]. It is reported that IGHG1 down regulation with siRNA silencing can suppress cell proliferation and apoptosis in prostate cancer cells [61]. In stroke patients, platelets are affected when inflammation markers are induced in plasma [27]. In the findings of this study, IGHG1 and IGHG3 are first determined in stroke patients’ platelets, these proteins may interact with platelets surface receptors. Furthermore, MPO is known as a heme protein and cellular catalytic enzyme which is released form monocyte and neutrophils. It has been shown that MPO binds to human platelets and triggers platelet activation and aggregation via releasing Ca^2+^ from platelets’ intracellular stores [62]. In addition, ALOX12 is a known arachidonate 12-lipoxygenase and its role in platelets functions is not well understood. It was previously reported that ALOX12 causes thrombin- and thromboxane-induced platelet aggregation which interferes with the calcium signaling in platelets [63]. In addition to this role of ALOX12 in platelets, it mediates the generation of peroxide and other reactive oxygen species which are regulated by the nicotinamide adenine dinucleotide phosphate oxidase pathway in platelets [64]. On the other hand, APOA1, apolipoprotein A-I, binds to the platelet surface and has an antithrombotic role in blood circulation. It binds to APOA1 to the scavenger receptor BI which regulates platelet function, most likely through platelet inhibition [65]. Our data showed that APOAI levels decreased in the platelets of stroke patients. In addition, APOH is called β2-Glycoprotein I (β2GPI) and is associated with thrombosis and causes thrombophilia [66]. In blood circulation, β2GPI may also be complex with ox-LDL (oxidative modification of low-density lipoprotein) and these complexes promote prothrombotic functions and coagulation factors in atherothrombotic cardiovascular diseases [67]. Platelets included the CD36 receptor that is required for the activation of human platelets in response to oxLDL. In a previous paper it was shown that through the addition of ox-LDL induced oxidative modifications and the triggering signals of apoptosis activation in platelets [68]. When considering the effects of APOH on stroke patient’s platelets, pro-apoptotic factors must be activated.

There is a paucity of studies focused on platelet functions in diseases. It is important to define and characterize platelet status in acute ischemic stroke patients in order to generate new anti-platelet drugs or to discover platelet activation markers for diagnosis. For the purposes of this study, we demonstrated that 83 proteins were differentially expressed in the stroke group in contrast to the control group. Some of these proteins have been suggested to play an important role in the activation of platelets and the pathophysiology of acute ischemic stroke. These findings may be instrumental in facilitating future mechanistic studies.

## Supporting Information

S1 Fig Distribution of localization of identified proteins. Classification of cellular compartment in stroke patients platelet compare to control group
(TIF)

S2 Fig Distribution of classification of identified proteins A) Platelet Biological process of stroke patients as compared to control group, B) Platelet functional categories of stroke patients as compared to control group. (TIF)

S1 Table Acute ischemic stroke patients and controls characteristic. (DOCX)

S2 Table Summary of proteins significantly up/down regulated in stroke vs. controls. (DOCX)
