# Supplementary figures and images for: Platelets Proteomic Profiles of Acute Ischemic Stroke Patients
Source: PLoS One. 2016 Jun 23;11(6):e0158287. doi: 10.1371/journal.pone.0158287 (PMC4919045; doi:10.1371/journal.pone.0158287)

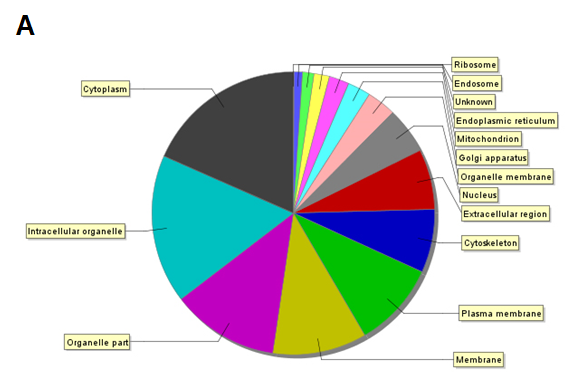

Supplement: S1 Fig — Classification of cellular compartment in stroke patients platelet compare to control group (TIF) [file pone.0158287.s001.tif]

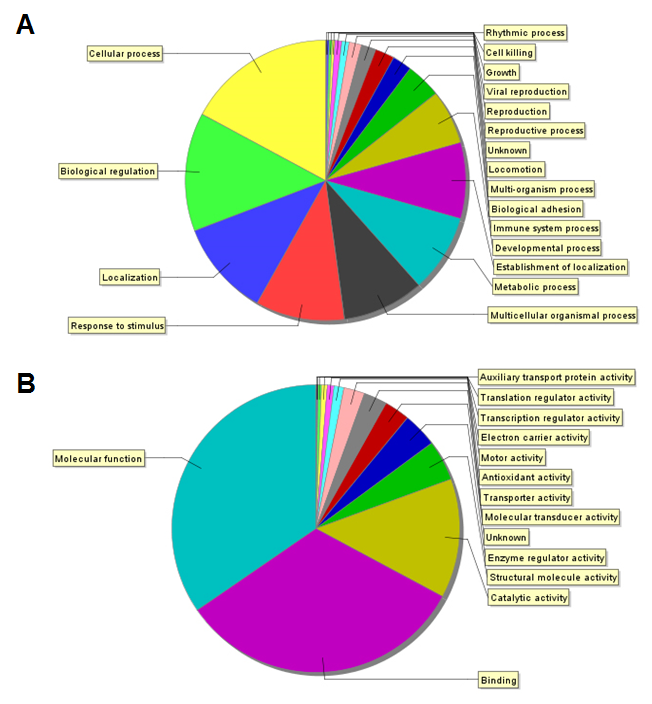

Supplement: S2 Fig — (TIF) [file pone.0158287.s002.tif]
