# Supplementary material for: Platelets Proteomic Profiles of Acute Ischemic Stroke Patients
Source: PLoS One. 2016 Jun 23;11(6):e0158287. doi: 10.1371/journal.pone.0158287 (PMC4919045; doi:10.1371/journal.pone.0158287)
Supplement: S1 Table — (DOCX) [file pone.0158287.s003.docx]

| **Acute ischemic stroke patients and controls characteristic** | | | |
| --- | --- | --- | --- |
| **Patients characteristics** | Stroke (n=65) | Controls (n=42) | P value |
| Age, years | 66.1±10.5 | 50.2±7.8 |  |
| Male sex | 68% | 53% |  |
| Alcohol % | 2% | NA |  |
| Hypertension % | 45% | NA |  |
| Diabetes mellitus % | 12% | NA |  |
| Dyslipidemia % | 38% | NA |  |
| Current smoker % | 10% | NA |  |
| CAD % | 8% | NA |  |
| **BMI** |  |  |  |
| BMI˂30 | 79 % | 92% |  |
| Obese (BMI≥30) | 21% | 8% |  |
| **Platelet characteristics** |  |  |  |
| PLT (10^3^/uL) | 112.74±65.38 | 258±82 | *** |
| MPV (fL) | 7.4±1.5 | 9.9±2.1 | * |
| PCT | 0.182±0.52 | 0.205±0.11 | ns |
| PDW | 14.20±6.23 | 18.75±6.02 | ns |
| BNP (pg/mL) | 78.07±20.2 | 152.09 ±16.41 | *** |
|  |  |  |  |

Abbreviations: BMI, Body Mass Index; BNP, brain natriuretic peptide; CAD, coronary artery disease; Data are given as average± standart deviation. NA, not applicable. Ns, non-significantly, *(p< 0.05), ***(p< 0.001)
