# Supplementary material for: Platelets Proteomic Profiles of Acute Ischemic Stroke Patients
Source: PLoS One. 2016 Jun 23;11(6):e0158287. doi: 10.1371/journal.pone.0158287 (PMC4919045; doi:10.1371/journal.pone.0158287)
Supplement: S2 Table — (DOCX) [file pone.0158287.s004.docx]

|  | | **Table. Summary of proteins significantly up/down regulated in stroke vs. controls** | | | | | |
| --- | --- | --- | --- | --- | --- | --- | --- |
| **Accession number** | **Description** | | **PLGS**  **Score** | **Fold change Stroke vs.Control** | **Functional Classification** | **Subcellular Localization** | **P value** |
| P61981 | 14 3 3 protein gamma | | 721.62 | 1.35 | Cell signaling | Cytoplasm | 0.18 |
| P60709 | Actin cytoplasmic 1 | | 17972.36 | 0.8 | Cell adhesion | Cytoplasm | 0.02 |
| P02763 | Alpha 1 acid glycoprotein 1 | | 281.74 | 0.32 | Transport | Extracellular Space | 0.08 |
| P01009 | Alpha 1 antitrypsin | | 408.1 | 0.34 | Hemostasis | Extracellular Space | 0.06 |
| P04217 | Alpha 1B glycoprotein | | 987.01 | 1 | Structural | Extracellular Space | 0.99 |
| Q08043 | Alpha actinin 3 | | 676.55 | 1.52 | Structural | Cytoplasm | 0.17 |
| O43707 | Alpha actinin 4 | | 2668.75 | 1.36 | Transport | Cytoplasm | 0.13 |
| P61163 | Alpha centractin | | 212.96 | 2 | ATP-binding | Cytoplasm | 0.71 |
| P01008 | Antithrombin III | | 574.46 | 1 | Hemostasis | Extracellular Space | 0.90 |
| P02647 | Apolipoprotein A I | | 13518.41 | 0.14 | Transport | Extracellular Space | 0.001 |
| P18054 | Arachidonate 12 lipoxygenase 12S type | | 119.44 | 2 | Cellular metabolic process | Cytoplasm | 0.011 |
| P02749 | Beta 2 glycoprotein 1 | | 505.83 | 1 | Coagulation | Extracellular Space | 0.010 |
| Q562R1 | Beta actin like protein 2 | | 5519.13 | 0.75 | Structural | Cytoskeleton | 0.09 |
| Q9UBW5 | Bridging integrator 2 | | 278.82 | 2 | Cell junction | Cytoplasm | 0.97 |
| P00450 | Ceruloplasmin | | 841.24 | 0.23 | Structural | Extracellular Space | 0.18 |
| P10909 | Clusterin | | 678.34 | 0.68 | Immunity | Extracellular Space | 0.009 |
| P01024 | Complement C3 | | 556.52 | 0.3 | Lipid metabolism  Immunity | Extracellular Space | 0.007 |
| P08603 | Complement factor H | | 364.85 | 1 | Immunity | Extracellular Space | 0.14 |
| P31146 | Coronin 1A | | 556 | 0.77 | Cellular response | Cytoplasm | 0.10 |
| P46109 | Crk like protein | | 189.3 | 1 | Cell signaling | Cytoplasm | 0.001 |
| Q9H223 | EH domain containing protein 4 | | 262.33 | 2 | ATP-binding | Plasma Membrane | 0.64 |
| P02671 | Fibrinogen alpha chain | | 721.32 | 0.92 | Cell adhesion | Plasma Membrane | 0.046 |
| P02675 | Fibrinogen beta chain | | 665.15 | 0.81 | Cell adhesion | Plasma Membrane | 0.082 |
| P02679 | Fibrinogen gamma chain | | 821.03 | 1 | Cell adhesion | Plasma Membrane | 0.074 |
| P06396 | Gelsolin | | 694.21 | 1.12 | Cytoskeleton | Cytoplasm | 0.061 |
| P00738 | Haptoglobin | | 1335.75 | 2.32 | Immunity | Extracellular Space | 0.06 |
| P00739 | Haptoglobin related protein | | 359.73 | 2.27 | Immunity | Extracellular Space | 0.12 |
| P54652 | Heat shock related 70 kDa protein 2 | | 1558.19 | 0.71 | Chaperone | Cytoplasm | 0.25 |
| P69905 | Hemoglobin subunit alpha | | 12788.05 | 5.26 | Transport | Cytoplasm | 0.057 |
| P68871 | Hemoglobin subunit beta | | 28866.22 | 2.41 | Transport | Cytoplasm | 0.08 |
| P02042 | Hemoglobin subunit delta | | 6847.57 | 3.97 | Transport | Cytoplasm | 0.22 |
| P04196 | Histidine rich glycoprotein | | 248.31 | 1 | Hemostasis | Extracellular Space | 0.001 |
| Q96KK5 | Histone H2A type 1 H | | 3480.2 | 2 | Structural | Nucleus | 0.90 |
| Q8N257 | Histone H2B type 3 B | | 2018.78 | 2 | Structural | Nucleus | 0.92 |
| P10316 | HLA class I histocompatibility antigen A 69 alpha chain | | 410.01 | 1 | Immunity | Plasma Membrane | 0.76 |
| P30462 | HLA class I histocompatibility antigen B 14 alpha chain | | 360.53 | 1 | Immunity | Plasma Membrane | 0.61 |
| P30501 | HLA class I histocompatibility antigen Cw 2 alpha chain | | 81.75 | 2 | Immunity | Plasma Membrane | 0.90 |
| P01876 | Ig alpha 1 chain C region | | 214.89 | 0.4 | Structural | Extracellular Space | 0.05 |
| P01857 | Ig gamma 1 chain C region | | 4185.43 | 0.81 | Structural | Extracellular Space | 0.004 |
| P01860 | Ig gamma 3 chain C region | | 1528.76 | 0.49 | Structural | Extracellular Space | 0.008 |
| P01766 | Ig heavy chain V III region BRO | | 517.92 | 0.23 | Immune response | Extracellular Space | 0.22 |
| P01779 | Ig heavy chain V III region TUR | | 238.66 | 1 | Immune response | Extracellular Space | 0.91 |
| P01764 | Ig heavy chain V III region VH26 | | 1136.17 | 1 | Immune response | Extracellular Space | 0.08 |
| P01834 | Ig kappa chain C region | | 4425.88 | 1.88 | Structural | Extracellular Space | 0.06 |
| P01596 | Ig kappa chain V I region CAR | | 460.87 | 2 | Immune response | Extracellular Space | 0.61 |
| P04206 | Ig kappa chain V III region GOL | | 1041.26 | 1 | Immune response | Extracellular Space | 0.57 |
| P08514 | Integrin alpha IIb | | 5865 | 2.8 | Cell adhesion | Plasma Membrane | 0.001 |
| P05106 | Integrin beta 3 | | 3757.28 | 3.76 | Cell adhesion | Plasma Membrane | 0.001 |
| Q14624 | Inter alpha trypsin inhibitor heavy chain | | 266.67 | 1 | Immunity | Extracellular Space | 0.29 |
| P19823 | Inter alpha trypsin inhibitor heavy chain H2 | | 457.67 | 0.21 | Unknown | Extracellular Space | 0.77 |
| Q9Y624 | Junctional adhesion molecule A | | 469.39 | 0.77 | Cell adhesion | Plasma Membrane | 0.16 |
| P01042 | Kininogen 1 | | 216.23 | 1 | Hemostasis | Extracellular Space | 0.19 |
| P00338 | L lactate dehydrogenase A chain | | 997.59 | 1.28 | Cellular metabolic process | Cytoplasm | 0.12 |
| Q14766 | Latent transforming growth factor beta binding protein 1 | | 230.07 | 0.71 | Cell signaling | Extracellular Space | 0.14 |
| P05164 | Myeloperoxidase | | 137.07 | 2 | Oxidative stress | Cytoplasm | 0.001 |
| P14649 | Myosin light chain 6B | | 2488.76 | 0.79 | Structural | Cytoplasm | 0.33 |
| P62937 | Peptidyl prolyl cis trans isomerase A | | 3669.04 | 0.79 | Cell adhesion | Cytoplasm | 0.07 |
| P05155 | Plasma protease C1 inhibitor | | 240.32 | 1 | Hemostasis  Immune response | Extracellular Space | 0.82 |
| P00747 | Plasminogen | | 140.71 | 1 | Hemostasis | Extracellular Space | 0.24 |
| P02776 | Platelet factor 4 | | 16060.84 | 0.76 | Chemotaxis | Extracellular Space | 0.05 |
| P13224 | Platelet glycoprotein Ib beta chain | | 2621.19 | 0.8 | Structural | Plasma Membrane | 0.05 |
| P14770 | Platelet glycoprotein IX | | 739.39 | 0.77 | Hemostasis | Plasma Membrane | 0.09 |
| Q6S8J3 | POTE ankyrin domain family member E | | 6488.06 | 0.41 | Ubiquitination | Extracellular Space | 0.23 |
| P0CG38 | POTE ankyrin domain family member I | | 1434.38 | 0.28 | unknown | unknown | 0.23 |
| P02760 | Protein AMBP | | 416.33 | 1 | Cell adhesion | Extracellular Space | 0.01 |
| Q15084 | Protein disulfide isomerase A6 | | 241.9 | 1.26 | Chaperone | Cytoplasm | 0.12 |
| P00734 | Prothrombin | | 180.17 | 1 | Hemostasis | Extracellular Space | 0.90 |
| Q9BYX7 | Putative beta actin like protein 3 | | 5395.38 | 2.27 | Structural | Cytoskeleton | 0.31 |
| Q92928 | Putative Ras related protein Rab 1C | | 1290.68 | 1.39 | Transport | Cytoplasm | 0.27 |
| Q99867 | Putative tubulin beta 4q chain | | 206.49 | 4.85 | Structural | Cytoskeleton | 0.47 |
| P14618 | Pyruvate kinase isozymes M1 M2 | | 2953.41 | 1.23 | Cellular metabolic process | Cytoplasm | 0.04 |
| P30613 | Pyruvate kinase isozymes R L | | 343.4 | 0.42 | Cellular metabolic process | Cytoplasm | 0.41 |
| Q15404 | Ras suppressor protein 1 | | 571.72 | 1.4 | Cell signaling | Cytoplasm | 0.001 |
| P52566 | Rho GDP dissociation inhibitor 2 | | 347.63 | 0.81 | Cell signaling | Cytoplasm | 0.17 |
| P16615 | Sarcoplasmic endoplasmic reticulum calcium ATPase 2 | | 167.85 | 1 | Transport | Cytoplasm | 0.94 |
| Q6ZU15 | Septin 14 | | 137.47 | 2 | Cell cycle | unknown | 0.26 |
| P07996 | Thrombospondin 1 | | 4873.67 | 1.4 | Cell adhesion | Extracellular Space | 0.001 |
| P61586 | Transforming protein RhoA | | 396.21 | 0.82 | Cell projection | Cytoplasm | 0.015 |
| Q9H4B7 | Tubulin beta 1 chain | | 4467.07 | 1.38 | Transport | Cytoplasm | 0.24 |
| P68371 | Tubulin beta 2C chain | | 1949.68 | 0.21 | Structural | Cytoplasm | 0.18 |
| Q9BUF5 | Tubulin beta 6 chain | | 1224.04 | 0.53 | Cytoskeleton | Cytoplasm | 0.32 |
| P62987 | Ubiquitin 60S ribosomal protein L40 | | 483.73 | 1 | Ubiquitination | Cytoplasm | 0.97 |
| P04004 | Vitronectin | | 728.01 | 1 | Cell adhesion | Extracellular Space | 0.001 |

Data indicates the abundance ratio for stroke and control groups. Statistical significance (p< 0.05) was determined using the mixed-effects linear method as described in materials and methods. The protein identification results for each triplicate are shown in table 2 (Stroke versus control). Indicated are the levels of confidence for protein identification (OK = 2: >95%; OK = 1: between 50% and 95%), the SwissProt accession number, entry and description, PLGS score, functional classification, subcellular localization number. ns: not significantly
